# Inhibition of MurA Enzyme from *Escherichia coli* and *Staphylococcus aureus* by Diterpenes from *Lepechinia meyenii* and Their Synthetic Analogs

**DOI:** 10.3390/antibiotics10121535

**Published:** 2021-12-15

**Authors:** Macarena Funes Chabán, Martina Hrast, Rok Frlan, Dafni G. Graikioti, Constantinos M. Athanassopoulos, María Cecilia Carpinella

**Affiliations:** 1 Fine Chemical and Natural Products Laboratory, IRNASUS CONICET-UCC, Universidad Católica de Córdoba, Córdoba 5016, Argentina; macarenafuneschaban@ucc.edu.ar; 2 Faculty of Pharmacy, University of Ljubljana, SI-1000 Ljubljana, Slovenia; martina.hrast@ffa.uni-lj.si (M.H.); Rok.Frlan@ffa.uni-lj.si (R.F.); 3 Synthetic Organic Chemistry Laboratory, Department of Chemistry, University of Patras, 26504 Patras, Greece; dafnigraikioti@upnet.gr (D.G.G.); kath@chemistry.upatras.gr (C.M.A.)

**Keywords:** dehydroabietane derivatives, diterpenes, MurA and MurF inhibitors, *Staphylococcus aureus* MurA, *Escherichia coli* MurA

## Abstract

Enzymes MurA and MurF, involved in bacterial cell wall synthesis, have been validated as targets for the discovery of novel antibiotics. A panel of plant-origin antibacterial diterpenes and synthetic analogs derived therefrom were investigated for their inhibitory properties on these enzymes from *Escherichia coli* and *Staphylococcus aureus*. Six compounds were proven to be effective for inhibiting MurA from both bacteria, with IC_50_ values ranging from 1.1 to 25.1 µM. To further mechanistically investigate the nature of binding and to explain the activity, these compounds were docked into the active site of MurA from *E. coli*. The aromatic ring of the active compounds showed a T-shaped π–π interaction with the phenyl ring of Phe328, and at least one hydrogen bond was formed between the hydroxy groups and Arg120 and/or Arg91. The results disclosed here establish new chemical scaffolds for the development of novel entities targeting MurA as potential antibiotics to combat the threat of pathogenic bacteria, particularly resistant strains.

## 1. Introduction

In the bid to fight pathogenic bacteria, medicine has developed a vast arsenal of antibiotics, which have extended the human lifespan [1]. However, the outbreak of resistant bacteria has led to failures in therapies, turning the treatment of infectious diseases into a serious global health threat [2,3]. Therefore, academic research and the industry face a challenge to find effective alternative antibiotics to tackle these serious infections.

The enzymes that participate in the biosynthesis of the bacterial cell wall, especially its essential component, peptidoglycan (PG), are attractive selective targets to develop agents against Gram-positive and Gram-negative pathogenic bacteria [4]. Since the main functions of PG are to maintain the integrity and shape of the bacterial cell and to participate in cell growth and division [4,5,6], the disruption of its synthesis will result in cell lysis that hinders bacterial survival [7,8]. The biosynthetic pathway of PG comprises of a multi-step process that takes place in three different stages: cytoplasmic, membrane-associated and periplasmic [6]. The PG synthesis in the cytoplasm involves the conversion of uridine diphosphate-*N*-acetylmuramyl-pentapeptide (UDP-MurNAc-pp) from uridine diphosphate-*N*-acetylglucosamine (UDP-GlcNAc) by enzymes MurA through MurF [5,6]. MurA transferase initiates the biosynthesis by transferring enolpyruvate from phosphoenolpyruvate (PEP) to UDP-GlcNAc yielding enolpyruvyl UDP-GlcNAc [9,10] while MurF ligase is involved in the final step of this pathway by introducing a dipeptide, usually D-Ala–D-Ala [8], into UDP-MurNAc-tripeptide [6]. In this context, Mur cascade enzymes represent advantageous targets for antibiotic discovery; however, interference in the activity of these is underexplored over other target sites also involved in PG synthesis [11]. It is worth noting that the inhibition of Mur enzymes represents a highly selective method in the battle against bacterial pathogens since there are no mammalian counterparts of these proteins [9]. The naturally occurring broad-spectrum antibiotic, fosfomycin, which acts as an analogue of PEP by covalently reacting with the active site Cys residue of MurA [9,12], is the unique inhibitor of this enzyme approved for clinical use [8]. However, resistance to fosfomycin, a phenomenon caused by various mechanisms, including the overexpression or mutations of MurA, leads to less effective therapies [8,13,14]. This scenario drives efforts to identify novel chemical scaffolds with inhibitory properties on Mur enzymes.

**Figure 1:**
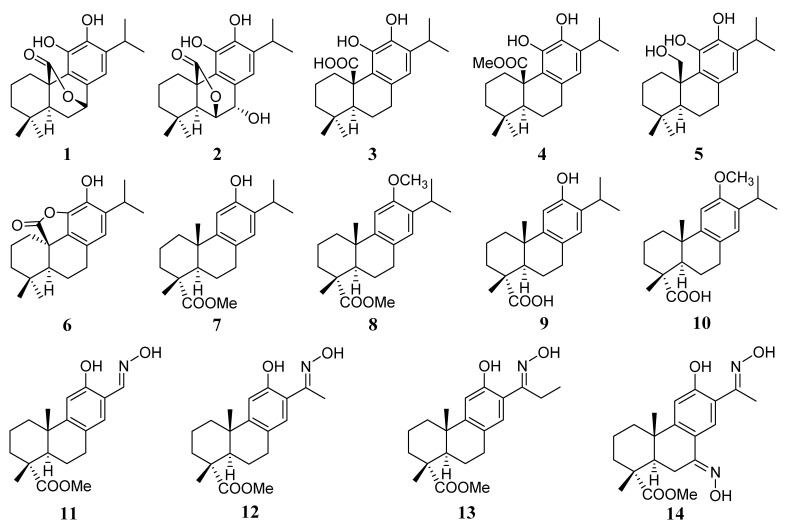
Chemical structures of the naturally occurring carnosol (**1**), rosmanol (**2**) and carnosic acid (**3**) as well as of the synthetic carnosic and dehydroabietic acid derivatives **4**–**14**.

The diterpenes isolated from *Lepechinia meyenii*, carnosol (**1**), rosmanol (**2**) and carnosic acid (**3**) [3], the derivatives thereof, 20-methyl carnosate (**4**) and carnosic acid-γ-lactone (**6**) [3], as well as the dehydroabietic derivatives **11**–**14** [15] (Figure 1), demonstrated promising antibacterial activity against several strains of methicillin- sensitive and resistant *Staphylococcus aureus* (MSSA and MRSA, respectively) [3,15].

Prompted by these results and aiming to determine whether the antibacterial properties of the diterpenes were related to the inhibition of Mur enzymes, the above-mentioned entities were tested for their ability to act as MurA and MurF inhibitors. The structure–activity relationships supported by molecular modelling analysis led to the design of new compounds, which were further evaluated. The present work provides a new avenue for the design of potential antibacterial drugs that interfere with the biosynthesis of PG.

## 2. Results

A panel of natural diterpenes and synthetic analogs (Figure 1), some of which were previously reported as encouraging antibacterial entities [3,15], were evaluated for their interfering properties on MurA and MurF enzymes.

In the first step, the residual activity (RA) of both enzymes was determined in the presence of the diterpenes. All the assayed compounds **1**–**14** failed to inhibit *E. coli* and *S. aureus* MurF as is evident from the RA values obtained ranging from 67 to 100%, even under pre-incubation conditions. These RA values were higher than 50%, the threshold value established as promising for conducting further studies [10]. When the compounds were tested against MurA from *E. coli* and *S. aureus* without pre-incubation, only compounds **1** and **4** showed RA values lower than 50% (Table 1). On the contrary, when the enzymes were pre-incubated with the tested diterpenes, compounds **1**–**6** and **9** showed RA values ranging from 0 to 28% (Table 1). Encouraged by these results, these compounds were subjected to further evaluation to determine their half-inhibitory concentrations (IC_50_).

As observed in Table 1, compound **4** was identified to efficiently inhibit MurA from *E. coli* and *S. aureus,* with both RAs corresponding to 0% and IC_50_ values of 2.8 and 3.4 µM, respectively. Compound **1** displayed RA values of 5 and 3%, respectively, with IC_50_ values also in the low micromolar range, corresponding to 2.8 and 1.1 µM, respectively (Table 1). On the other hand, compounds **2**, **3**, **5** and **6** showed different levels of activity with IC_50_ values ranging from 4.8 to 25.1 µM (Table 1). Although compound **9** showed 23 and 28% of RA on MurA from *E. coli* and *S. aureus*, respectively, the corresponding IC_50_ values of 49.4 and 55.2 µM revealed very weak activity. Compounds **7**, **8** and **10**–**14** showed no inhibitory properties on both MurA enzymes, with RA values ranging from 72 to 100% (Table 1). Table 1 show that compounds are time-dependent inhibitors.

According to the results obtained, compounds **1**, **4** and **6** showed significant inhibition of MurA derived from *E. coli* displaying IC_50_ values less than 5 µM (Table 1). However, these results contrasted with the inability of these molecules to affect the development of *E. coli*, as was evidenced by the minimum inhibitory concentration (MIC) values higher than 250 µg/mL, a value four-times higher than the MIC established to consider a compound as promising antibacterial [3]. The lack of activity could be attributed to poor penetration of the diterpenes into the bacterial cell. When the membrane perturbing antibiotic polymyxin B, was added at sub-inhibitory concentration, *E. coli* became susceptible to compounds 1, 4 and 6 with MIC values of 125, 3.9 and 125 µg/mL, respectively. Vancomycin, used as a positive control, showed a MIC value of 250 µg/mL while the addition of polymyxin B decreased this value to 62.5 µg/mL. As noted, in combination with the latter, compounds **1** and **6** showed similar activity to that observed with vancomycin, whereas compound **4** was more effective.

**Figure 2:**
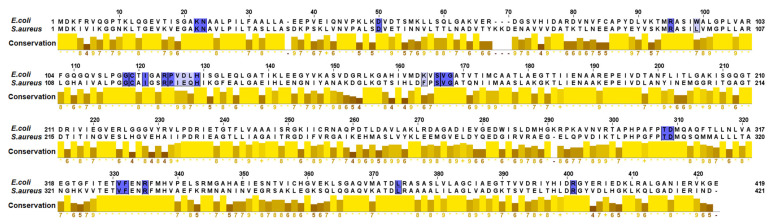
Conservation of amino acids (colored blue) within 4 Å from the PEP and UDP-GlcNAc binding sites in *Escherichia coli* and *Staphylococcus aureus* MurA enzymes.

To gain a better understanding of the enzyme–ligand interactions, a computational analysis of inhibitors **1**–**6** and of the inactive compounds was undertaken using the available crystal structures of MurA from *E. coli* in the form of binary or ternary complexes (PDB codes in the Protein preparation section under Materials and Methods). Comparison of the amino acid sequences in the active sites of MurA from *E. coli* and *S. aureus* showed 100% conservation of residues that are near the PEP binding site where the catalytic Cys is located. A slight variability was observed in the UDP-GlcNAc binding site, mostly within the residues that form interactions with uracil. A variability in residue 95 that forms hydrophobic interactions with glucosamine was also observed. While in *E. coli,* residue 95 was occupied with Trp, in *S. aureus* this position was occupied with Leu (Figure 2).

**Figure 3:**
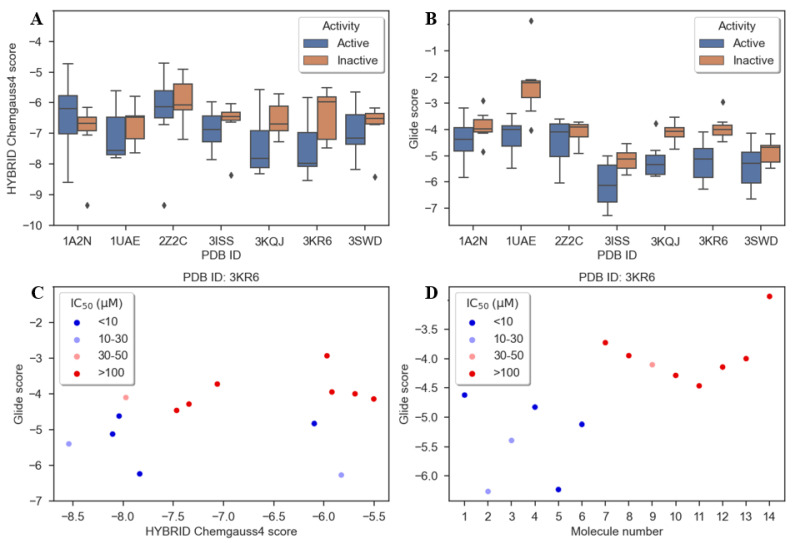
Docking results. (**A**) and (**B**) Boxplots of Hybrid and Glide docking scores of active and inactive compounds calculated for each enzyme separately; (**C**) scatterplot of Glide and Hybrid scores for each compound performed for the enzyme with PDB ID: 3KR6; (**D**) Glide scores per each compound for enzyme with PDB ID: 3KR6. IC_50_ values in labels are inhibitory concentrations on MurA from *E. coli*.

Docking analysis was then performed using the docking programs Shrodinger’s Glide [16] and OpenEye’s Hybrid [17]. All seven crystal structures of MurA from *E. coli* previously deposited in the RCSB Protein Data Bank were used. The results obtained from both docking programs applied on the seven crystal structures were carefully analyzed to select the most appropriate structure and the program that could distinguish better between active (IC_50_ < 25 μM) and weak or inactive compounds (IC_50_ > 45 μM) and therefore explain the biological findings (Figure 3, Figures S1 and S2 in Supplementary Materials). Interestingly, large differences in the distribution of docking scores were obtained for each crystal structure of *E. coli* MurA (Figures S1 and S2). In addition, Glide provided slightly better results in terms of its ability to distinguish between active and inactive entities and was therefore used for further analysis of the docking poses (Figure 3A,B). Among the seven crystal structures, only 1UAE, 3KQJ and 3KR6 provided docking scores that could distinguish reasonably well between both groups of molecules, using Glide. After visual inspection of the scatterplots with the Glide scores for each molecule, 3KR6 was finally selected as the most suitable crystal structure (Figure 3C,D).

**Figure 4:**
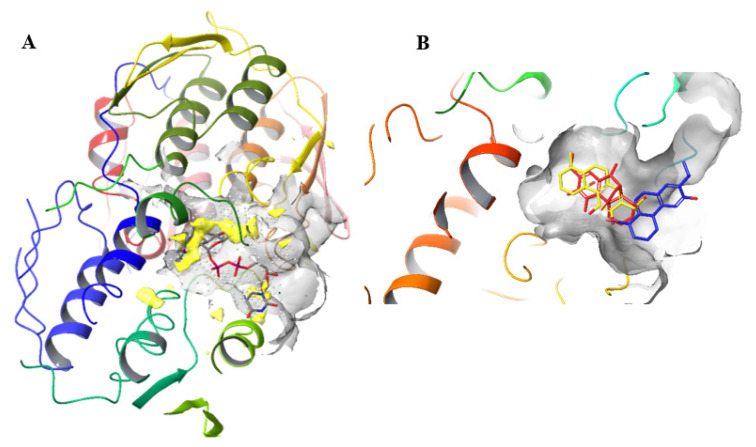
(**A**) Binding mode of UDP-GlcNAc. Hydrophobic areas of binding cavities are colored as yellow solid; (**B**) Differences in the binding modes of the active compounds **1** (colored red) and **4** (colored yellow) and of the inactive compound **10** (colored blue).

The binding mode of UDP-GlcNAc in MurA is presented in Figure 4A. The protein structure is shown as a colored ribbon diagram, and UDP-GlcNAc is shown as a stick presentation. The hydrophobic region of the enzyme surrounding the ligand is colored as a yellow solid surface which was calculated with SiteMap [16]. As shown in Figure 4A, the MurA enzyme is folded into two globular domains (N- and C-terminal domains) connected by two linker regions. UDP-GlcNAc and PEP bound to the cleft formed between both domains and upon ligand binding are closed by a flexible ten amino acid lid of the N-terminal domain. Although both substrates are highly polar, this ligand-binding region contains two larger hydrophobic patches (colored yellow) near the regions where the glucosamine and uridine part of UDP-GlcNAc bind [18,19].

The binding mode of typical representatives of the active and inactive compounds is shown in Figure 4B. The majority of active compounds **1**–**6** that docked to MurA occupied the UDP-GlcNAc binding pocket and adopted a conformation in which the phenolic part of the molecule was directed toward the interior of the enzyme, and the saturated distal cyclohexyl ring was directed toward the exterior of the enzyme. Compound **4** was an exception among the active compounds. In the binding mode of this compound, the structure was rotated by 180°, but the geometric position of the phenolic OH group on C-11, which forms a hydrogen bond with Arg91, remained the same as in all other active molecules. As expected, for compound **9**, Glide predicted no interaction with residues within the enzyme but rather positioned itself at the entrance of the UDP-GlcNAc binding site, which would explain the weak activity of this diterpene. Its binding mode was therefore very similar to the binding modes of the inactive oximes **11**–**14**, all of which formed weaker interactions with residues at the entrance to the binding site.

**Figure 5:**
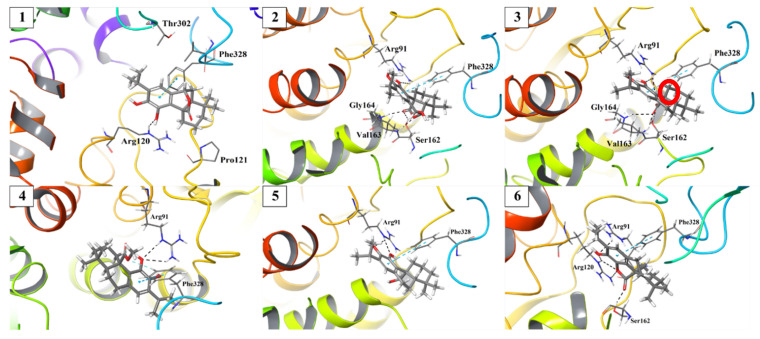
Binding modes of compounds **1**–**6**.

A detailed 3D representation of the highest-ranking docking modes of compounds **1**–**6** is presented in Figure 5. The amino acids surrounding the ligand and forming non-covalent interactions are shown as rods; the protein structure is shown as a colored ribbon. Analysis of the predicted binding mode showed that all active compounds **1**–**6** form a T-shaped π–π interaction between the aromatic ring of the diterpenes and the phenyl ring of Phe328. Moreover, at least one hydrogen bond was formed between OH groups on C-11 and/or C-12 and Arg120 (compounds **1** and **6**) or Arg91 (compounds **2**–**6**). Compounds **2**, **3** and **6**, showed to be effective by the formation of additional hydrogen bonds between the carboxyl (compound **3**) or the γ-lactone (compounds **2** and **6**) moieties and residues Ser162, Val163 and Gly164 located in the biphosphate binding region of the enzyme.

## 3. Discussion

As previously reported, a promising activity against MSSA and MRSA was determined for a panel of plant-origin and synthetic dehydroabietanes [3,15]. Based on these results, further investigations were carried out to establish whether the antibiotic potential of these molecules was related to the inhibition of Mur enzymes. This correlation was evident for compounds **1**–**4** and **6** against MurA from *E. coli* and *S. aureus*, which position this enzyme as a relevant target of these diterpenes. Compound **4** (Figure 1) proved to be the most potent inhibitor of the growth of the panel of MSSA and MRSA strains assayed, with MICs ranging from 1.9 to 7.8 µg/mL [3]. Likewise, this compound showed an antibacterial effect against *E. coli* in combination with polymyxin B. The addition of polymyxin B may overcome the cell membrane problem, allowing compound **4** to cross this barrier and therefore reducing the MIC values from >250 to 3.9 µg/mL. It is important to highlight that compound **4** reached the same MIC value against *E. coli* as those observed against different strains of MSSA and MRSA [3], which shows its promising antibacterial activity, being equally effective against Gram-positive bacteria and the more resistant, due to the morphological characteristics, Gram-negative bacteria [20,21]. Although the known permeabilized effect of polymyxin B, for which it is widely used [22,23], the presence of another synergistic mechanism with compound **4** could not be discarded. The antibiotic effect of compound **4** is consistent with the inhibitory properties on *E. coli* and *S. aureus* MurA with IC_50_ values of 2.8 and 3.4 µM, respectively (Table 1). The obtained results suggest that the inhibition of the transferase could be the primary target of compound **4**. The second most active antibacterial entity was compound **12**, with MIC values ranging from 3.9 to 15.6 µg/mL against MSSA and MRSA [15]. However, this oxime was completely inactive against Mur enzymes, suggesting that it achieves its antibacterial activity through a different pathway. These findings are in agreement with the differences observed in the growth curves of MSSA and MRSA treated with compounds **4** and **12**. A delay in the growth rate of both microorganisms till 12–15 h was observed in treatments with the first diterpene, while compound **12** reduced the growth rate up to 6 h [3,15]. Further assays should be performed to elucidate the underlying mechanism of this molecule. Compound **3** showed to effectively inhibit MSSA and MRSA growth with MICs ranging from 7.8 to 15.6 µg/mL [3] and displayed an interesting inhibitory activity on MurA from *S. aureus* (IC_50_ = 12 µM), although it was less effective than compounds **1**, **2**, **4**–**6**. This moderate inhibition suggests that this is not the main mechanism associated with its antibacterial activity on the Gram-positive bacteria. In relation to this, Álvarez-Martínez and co-workers [24] mentioned that the probable mechanism for achieving the anti-MRSA activity of compound **3** would involve cell membrane rupture. The IC_50_ value of 25 µM on *E. coli* MurA makes it not worthwhile to study the effect of compound **3** on permeabilized *E. coli*. Compound **1,** and to a lesser extent, compound **6**, showed strong anti-MurA activity. However, their growth inhibitory effect against *E. coli* and *S. aureus* was not prominent (MIC value of 125 µg/mL against permeabilized *E. coli* and MIC values ranging from 15.6 to 62.5 µg/mL against the different strains of MSSA and MRSA assayed) [3]. These results suggest that other factors present in the whole cells might negatively affect the antibacterial effect.

According to the results obtained and in agreement with their antibacterial activity [3], the presence of OH-7 rendered compound **2** less effective for inhibiting MurA than compound **1** (*p* < 0.05). Previous results indicated that the presence of a lactone moiety in some sesquiterpene lactones is a prerequisite for obtaining antibacterial and anti-MurA activities [25], which is in agreement with that observed with compounds **1**, **2** and **6**. The latter showed more potent activity with respect to the ring-opened compound **3**, with a carboxylic acid at C-20 (Figure 1). As previously observed by Mendgen and co-workers [25], the acidic function weakens the anti-MurA effect, in accordance with the lower activity observed for compound **3**. The C-20-OH-11 lactonized compound 6 was less effective than the C-20-OH-7 lactone **1**, the latter showing a similar activity to that of compound **4** bearing a C-20 methyl ester.

To the best of our knowledge, the inhibitory properties of diterpenes on MurA have not been previously reported, which means that this is the first time that this property has been described in this family of compounds. The IC_50_ values obtained for compounds **1**–**6** are in agreement with the IC_50_ values, ranging from 0.2 to 8.8 µM, obtained for other MurA inhibitors, which have been considered promising molecules [10]. The fact that Gram-negative MurA enzymes, in particular that from *E. coli*, are considered more efficient than those from Gram-positive bacteria [26], highlights the inhibitory properties of the effective compounds on *E. coli* MurA. These results are very encouraging, especially taking into account that few MurA inhibitors have been developed in the last decade [10]. The significant presence of compounds **1**–**3** in the edible plant *Rosmarinus officinalis* [3,27] as well as the antimutagenic activity of compounds **1** and **3** [28] and the lack of undesired effect against the nematode *Caenorhabditis elegans* of the latter compound [29], suggest that these diterpenes lack of toxic effects. The fact that compounds **1**–**6** are devoid of activity against MurF would reveal a specific activity of these diterpenes.

As observed in Figure 5, compounds **1**–**6** interacted by hydrogen bonds with Arg120, Arg91, Ser162, Val163 and Gly164, which are conserved in *E. coli* and in *S. aureus* MurA (Figure 2). This similarity would explain the match between the IC_50_ values obtained in the treatments of both MurA enzymes with the mentioned compounds. The time-dependent inhibition observed in the treatments with compounds **1**–**6**, implies that these could act as slow-binding inhibitors, form covalent adducts or could behave as non-covalent suicide inactivators [30]. Although a covalent binding to Cys115 of *E. coli* and *Pseudomonas aeruginosa* MurA was first described for some sesquiterpene lactones [31], subsequent studies failed to confirm this irreversible binding [32]. In fact, the analysis of the crystal structure of *E. coli* MurA in complex with the sesquiterpene lactone cnicin and with UDP-GlcNAc, indicated a non-covalent suicide inhibition [32], which would be the mechanism of inhibition observed for compounds **1**–**6**. This statement is not only sustained by literature data [25,33], but also by the absence of Michael acceptors in the chemical structure of the diterpenes which are necessary for covalent interactions with Cys115. The binding of the active compounds to Arg120 and Arg91 is of great importance due to the key role of these amino acids in stabilizing the closed conformation of the enzyme through interaction with the phosphonate group of UDP-GlcNAc [9,34]. One of the mechanisms of fosfomycin resistance is the mutation of Cys115 to Asp, which rendered bacteria completely insensitive to the antibiotic [35]. This phenomenon is also the cause of the innate resistance to fosfomycin of some bacterial strains, including *Mycobacterium tuberculosis* and *Chlamydia trachomatis* [36], whereas Arg120 and Arg91 are well conserved in these and in other bacteria [26,37,38]. Therefore, the different mechanism for the inactivation of MurA by the dehydroabietane compounds, particularly in relation to the interactions with Arg120 and Arg91 and not involving the interaction with the thiol group of the Cys115, would minimize cross-resistance and would show efficacy against various bacteria, including *M. tuberculosis*. The latter is considered a high priority microorganism in the search for alternative antibiotics [39].

## 4. Materials and Methods

### 4.1. Reagents

Polymyxin, UDP-GlcNAc, D-Ala-D-Ala, PEP, ATP and Hepes were purchased from Sigma-Aldrich Co. (St Louis, MO, USA). UDP-MurNAc-L-Ala-D-Glu-m-DAP was obtained from BACWAN facility, School of Life Sciences, The University of Warwick, UK. MurA from *E. coli* and *S. aureus* were recombinant, expressed in *E. coli* [10], while MurF from *E. coli* and *S. aureus* were recombinant expressed in *E. coli* [40,41].

### 4.2. Synthesis of Carnosic and Dehydroabietic Acid Derivatives

The procedures for the synthesis of compounds **4**, **6**–**8** and **11**–**14** were performed as previously reported [3,15]. The synthesis of compounds **5**, **9** and **10** were carried out as described below. NMR spectra of the compounds are available in Supplementary Materials.

**11,20-dihydroxyferruginol (5).** Commercially available carnosic acid (3) at 70% purity (40 mg, 0.12 mmol), was dissolved in THF (0.04 mL) and borane dimethyl sulfide complex solution (0.1 mL, 1 mmol) was added dropwise at 0 °C. The mixture was stirred at room temperature under an argon atmosphere for 48 h. Then, it was cooled at 0 °C and the excess of borane dimethyl sulfide complex was quenched by the dropwise addition of 5% aqueous citric acid. The mixture was extracted with ethyl acetate (AcOEt) and the organic phase was washed successively with 5% aqueous citric acid, water and brine, dried over anhydrous Na_2_SO_4_ and concentrated to dryness under vacuum. The desired alcohol 5 was afforded as a crystalline white solid (8 mg, 31% yield) after flash column chromatography (FCC) purification using hexane/AcOEt 9:1 as eluent. R_f_: hexane/AcOEt 8:2 = 0.29. ^1^H NMR (CDCl_3_, 600.13 MHz,) δ: 6.53 (s, 1 H, H-14), 5.97 (broad s, 1 H, OH-20), 4.50 (d, *J* = 9.6 Hz, 1 H, OH-11), 3.97 (d, *J* = 10.2 Hz, 1 H, OH-12), 3.25 (m, 1 H), 3.22 (unresolved sept, 1 H, H-15), 2.86 (m, 2 H), 1.60–1.80 (m, 4 H), 1.52 (m, 1 H), 1.43 (m, 2 H), 1.30 (m, 2 H), 1.26 (s, 1 H), 1.22–1.24 (2 d, 6 H, CH_3_-16 and CH_3_-17), 0.98 (s, 3 H, CH_3_-18), 0.89 (s, 3 H, CH_3_-19) (Figure S3); ^13^C NMR (CDCl_3_, 150.9 MHz,) *δ*: 142.17, 142.06, 132.40, 129.96, 127.47, 118.89, 67.31, 52.66, 43.93, 41.02, 33.86, 33.56, 31.93, 31.26, 27.13, 22.77, 22.52, 22.26, 18.94, 18.89 (Figure S4); ESI-ITMS(-) *m*/*z*: 317.26 [C_20_H_30_O_3_]^−^, 675.48 [2M + K^+^]^−^ (Figure S9).

**12-hydroxy-dehydroabietic acid (9).** A solution of BBr_3_ 1.0 M in dichloromethane (CH_2_Cl_2_) (0.09 mL, 0.09 mmol) was added dropwise to a precooled at 0 °C solution of acid 10 (10 mg, 0.030 mmol) in dry 1,2-dichloroethane (0.15 mL). The mixture was stirred for 30 min at 0 °C and then for a further 3 h at room temperature. Upon completion, the solution was diluted in CH_2_Cl_2_ and washed with 5% aqueous citric acid, water and brine. The organic phase was collected, dried over anhydrous Na_2_SO_4_ and concentrated to dryness under vacuum. The product 9 was afforded (3.5 mg, 37% yield) as a yellow oil after FCC purification using Toluene/AcOEt 9:1 as eluting system. R_f_: PhMe/AcOEt 8:*2* = 0.34. ^1^H NMR (CDCl_3_, 600.13 MHz,) *δ*: 6.83 (s, 1 H, H-11), 6.62 (s, 1 H, H-14), 3.10 (sept, *J* = 6.6 Hz, 1 H, H-15), 2.79–2.85 (m, 2 H, H-7 and H-7′), 2.21 (dd, *J* = 12.6, 2.4 Hz, 1 H, H-6), 2.20 (d, *J* = 13.2 Hz, 1 H, H-6′), 1.70–1.87 (m, 5 H, H-1,1′,3,3′, 5), 1.47–1.54 (m, 2 H, H-2,2′), 1.27 (s, 3 H, CH_3_-10), 1.21 (s, 3 H, CH_3_-4), 1.24 and 1.22 (2 d, *J* = 6.6 Hz, 3 H each, CH_3_-16 and CH_3_-17) (Figure S5); ^13^C NMR (CDCl_3_, 150.9 MHz,) *δ*: 183.73, 150.72, 147.76, 131.71, 127.05, 126.73, 110.79, 47.35, 44.56, 37.90, 36.84, 36.64, 29.23, 26.81, 24.99, 22.72, 22.50, 21.87, 18.51, 16.25 (Figure S6); ESI-ITMS(-) *m*/*z*: 315.24 [C**_20_**H**_28_**O**_3_**]**^−^**, 631.33 [2M − H]^−^ (Figure S10).

**12-methoxy-dehydroabietic acid (10).** To a solution of methyl ester 8 (39 mg, 0.113 mmol) in dry DMSO (2.7 mL), under argon atmosphere, *tert*-BuOK (49 mg, 0.434 mmol) was added and the mixture was vigorously stirred at room temperature for 24 h. The mixture was poured into 4 mL of a HCl (aq) 0.5 M and stirred for 5 min. The resulting mixture was placed in a separatory funnel and extracted four times with diethyl ether (Et_2_O). The combined organic layers were washed successively with water and brine, dried over anhydrous Na_2_SO_4_ and concentrated to dryness under vacuum. The product 10 was afforded as white solid (30 mg, 81% yield) after FCC purification using Hexane/AcOEt 8:2 as eluent. R_f_: Hex/AcOEt 8:2 = 0.15. ^1^H NMR (CDCl_3_, 600.13 MHz,) δ: 6.84 (s, 1 H, H-11), 6.71 (s, 1 H, H-14), 3.80 (s, 3 H, OCH_3_-12), 3.22 (sept, *J* = 7.2 Hz, 1 H, H-15), 2.79–2.93 (m, 2 H, H-7 and H-7′), 2.29 (d, *J* = 13.2 Hz, 1 H, H-6), 2.25 (dd, *J* = 1.8, 12.6 Hz, 1 H, H-6′), 1.71–1.88 (m, 5 H, H-1,1′,3,3′, 5), 1.51–1.57 (m, 2 H, H-2,2′), 1.28 (s, 3 H, CH_3_-10), 1.24 (s, 3 H, CH_3_-4), 1.19 and 1.18 (2 d, *J* = 6.6 Hz, 3 H each, CH_3_-16 and CH_3_-17) (Figure S7); ^13^C NMR (CDCl_3_, 150.9 MHz,) *δ*: 184.23, 155.06, 147.17, 134.49, 126.64, 126.49, 106.33, 55.57, 47.43, 44.67, 37.97, 37.16, 36.66, 29.29, 26.44, 25.01, 22.88, 22.64, 21.90, 18.56, 16.24 (Figure S8); ESI-ITMS(-) *m*/*z*: 329.27 [C_21_H_30_O_3_]^−^, 659.17 [2M − H]^−^ (Figure S11).

### 4.3. Bacterial Isolates and Cultures

*E. coli* (Migula) Castellani and Chalmers (ATCC 25922) was used to determine the antibacterial effect of the most potent *E. coli* MurA inhibitors alone, or in combination with, polymyxin B. MacConkey agar was used as the culture media. Overnight subcultures of the test organism carried out on plate count agar medium (PCA) were used.

### 4.4. Antibacterial Susceptibility Testing

To analyze whether the absence of an antibacterial effect against *E. coli* of the potent anti-*E. coli* MurA compounds, **1**, **4** and **6**, was due to their inability to enter into the cells, the study of the antibacterial susceptibility was performed by adding the classic permeability enhancer, polymyxin B. MIC determinations were carried out by broth microdilution method as previously described [3,15] with the administration of the target compounds alone at 250 µg/mL or at 1.9–250 µg/mL with polymyxin B at half of the MIC (0.49 µg/mL) in Mueller–Hinton broth. After 24 h incubation at 37 °C with continuous shaking, absorbance was measured with an iMark micro-plate reader (Bio-Rad, Hercules, CA, USA) at 655 nm. Compound **1** was dissolved in DMSO, while compounds **4** and **6** were dissolved in ethanol or acetonitrile (ACN), respectively. Negative controls containing dissolution solvents at a final concentration of 2% or polymyxin B at 0.49 µg/mL were simultaneously carried out, and the growth was compared to the viability control, which contained only a culture medium. In order to demonstrate that permeabilization is occurring, vancomycin, whose target is the PG precursor lipid II present in *E. coli* [42], was used as a control [43]. The large size of this glycopeptide antibiotic avoids its entrance into Gram-negative bacteria [44], while the addition of polymyxin B overcomes this inconvenience [45].

### 4.5. MurA and MurF Inhibition Assays

MurA inhibition was determined with the colorimetric malachite green method in which the release of orthophosphate generated during the reaction is measured [10,46]. Briefly, 2.5 µL of each tested compound previously dissolved in DMSO at a concentration of 100 µM, were added in duplicate to 50 µL of the reaction mixture containing 50 mM HEPES pH 7.8, 0.005% Triton X-114, 200 µM UDP-GlcNAc, 100 µM PEP and purified MurA (200 nM) diluted in 50 mM Hepes at pH 7.8. The 50 µL reaction mixture for MurF inhibitory assay contained 50 mM Hepes pH 8.0, 50 mM MgCl_2_, 0.005% Triton X-114, 600 µM D-Ala-D-Ala, 100 µM UDP-MurNAc-L-Ala-D-Glu-*m*-DAP, 500 µM ATP and purified MurF (8 nM). Time-dependent inhibition assays were also performed. MurA was preincubated with substrate UDP-GlcNAc and target compounds for 10 min at 37 °C, and then the reaction was started by the addition of the second substrate PEP, resulting in a mixture with a final volume of 50 µL as above described. MurF was preincubated with compounds for 10 min at 37 °C, and the reaction was started by the addition of the above-mentioned substrates to get a mixture with a final volume of 50 µL, as already described. In both experiments, after incubation for 15 min at 37 °C, the reaction was stopped by adding Biomol**^®^** reagent (100 µL, Enzo Life Sciences, Inc., Farmingdale, NY, USA). The absorbance was measured at 650 nm after 5 min in a microplate reader (Synergy H4). The final concentration of DMSO in the reaction mixture was 5% (*v*/*v*). Fosfomycin was used as a reference in MurA assay. The percentage of RA was calculated in comparison to negative controls containing only 5% DMSO. The IC_50_ values, the concentration of the compound at which the residual activity was 50%, were determined by measuring the residual activities at seven different compound concentrations. The results are expressed as mean ± standard error.

### 4.6. Preparation of Ligands

The molecules were drawn with ChemDraw 18, and OpenBabel [47,48] was used to transform the structures into the SMILES format.

Ligands were prepared on the LigPrep module of Maestro software (v. 2021-3) [49]. LigPrep is a robust collection of tools designed to prepare high quality, low-energy 3D structures for large numbers of drug-like molecules, starting with 2D or 3D structures in Spatial Data File (SDF) or Maestro format. Correct protonation states were generated on the Epik module of Maestro software [50] at pH 7.0 ± 2.0. Energy minimization was performed using OPLS3 force field, and finally, tautomers were enumerated. The resulting structures were saved in Maestro format and were used further in docking procedures with Hybrid or Glide.

### 4.7. Comparison between the Active Sites of MurA

Amino acid sequences of MurA from *E. coli* and *S. aureus* were compared with the Clustal algorithm in Jalview v. 2.11.1.4. [51]. Crystal structure of MurA from *E. coli* (PDB ID: 3KR6) was first downloaded from the RCSB Protein Data Bank (PDB, https://www.rcsb.org/, accessed on 23 September 2021). The amino acids that were distanced within 6 Å from the UDP-GlcNAc and the inhibitor fosfomycin, which was bound in the PEP binding site, were selected and used as a reference for further alignment with Jalview. The amino acid sequence of *S. aureus* was downloaded as a FASTA sequence from Uniprot (Entry Q931H5) and aligned in Jalview to the amino acid sequence of *E. coli*.

### 4.8. Protein Preparation

Crystal structures of MurA (PDB ID: 1UAE [52], 3KQJ [53], 3KR6 [54], 3ISS [55], 3SWD [56], 1A2N [57], 2Z2C [58] were downloaded from the RCS PDB website [59] and prepared with the Protein Preparation Wizard module that was implemented into the Schrödinger Suite (Schrödinger Suite 2021-3) [60,61]. To keep regions between protein monomers from generating unphysical sites that score well but exist only in the crystal lattice and not in the solution, only monomer chain A was kept. In each PDB structure, ligands, waters and other co-crystallized agents were deleted. Bond orders were automatically assigned, hydrogens were added, selenomethionines were converted to methionines, missing side chains were added, ligands were removed, disulfide bridges were created if possible, waters beyond 5 Å radius from heteroatoms were removed, and heteroatoms were protonated at pH 7.0.

Finally, proteins were minimized using restrained minimization and were further used for grid box generation and subsequent docking procedures with Hybrid or Glide.

### 4.9. Molecular Modeling

Hybrid (OEDOCKING v 3.4.0.2.) [17,62] and Glide (Schrödinger Release 2021-3) [16,63] docking programs were used for docking analysis. For each docking program, grid boxes had to be prepared separately for each crystal structure.

### 4.10. Docking with Hybrid

The OpenEye Make receptor software (OpenEye Scientific Software) was applied to define the active site that was used in the docking procedures with OpenEye’s Hybrid molecular docking program (OEDOCKING 3.4.0.2.: OpenEye Scientific Software, Santa Fe, NM, USA, http://www.eyesopen.com, accessed on 23 September 2021) [17,62]. The search space was defined as a grid box enclosing the active site with dimensions 22.7 Å × 17.3 Å × 21.7 Å (volume of 8495 Å) [28]. The site shape potential was set with the inner contour disabled and the outer contour 1296 Å [28]. After the docking procedure was finished, docking results were saved as an sdf file for further analysis.

### 4.11. Docking with Glide

The Receptor Grid Generation module that was implemented into the Schrödinger Suite (Schrödinger Suite 2021-3) was used to define the grid box [60,61]. To soften the potential for nonpolar parts scaling factor of 1.0, a partial charge cutoff of 0.25 was used. The search space was defined as a grid box enclosing the active site centroid of UDP-GlcNAc and set to dock ligands with a similar size to the native ligand. Glide was then used for ligand docking in which flexible ligands, nitrogen inversions and ring conformations were sampled. Then the post-docking minimization docking results were saved as an sdf file for further analysis.

### 4.12. Generation of Figures

The graphical presentation of the three-dimensional structures of docking results was completed using Maestro software (v. 2021-3) [49]. Boxplots and stackplots were generated using the Seaborn [64] and Matplotlib [65] libraries in Jupyter Notebook [66].

## 5. Conclusions

In previous works, the growth inhibitory activity against different strains of pathogenic bacteria by select natural and synthetic dehydroabietane compounds (compounds **1**–**3** and compounds **4**, **6**–**8** and **11**–**14**, respectively) was studied [3,15]. The promising anti-MurA effect demonstrated for compounds **1**–**4** and **6** may explain their antibacterial action, with MurA acting as a molecular target. These results provide the first evidence for this biological property in compounds belonging to the diterpene family. The SAR study and docking analysis of compounds **1**–**14** shed light on the interaction pattern of these compounds with MurA, showing that the aromatic ring and the hydroxyl groups play a pivotal role. The valuable information obtained could establish the chemical bases for the design of alternative antibiotics with MurA inhibitory properties.

## Figures and Tables

**Table 1:** Inhibitory effects of compounds **1**–**14** on MurA enzyme.

Compounds	RA % (IC_50_ µM) *Escherichia coli* MurA ^a^	RA % (IC_50_ µM) *Escherichia coli* MurA ^b^	RA % (IC_50_ µM) *Staphylococcus aureus* MurA ^a^	RA % (IC_50_ µM) *Staphylococcus aureus* MurA ^b^
1	38 ± 2 (66 ± 8)	5 ± 1 (2.8 ± 0.7)	32 ± 3 (61 ± 7)	3 ± 1 (1.1 ± 0.8)
2	72 ± 2	9 ± 2 (12.9 ± 3.4)	70 ± 3	8 ± 1.5 (5.7 ± 2.1)
3	74 ± 4	8 ± 2 (25.1 ± 6.5)	72 ± 3	7 ± 1 (12.3 ± 2.5)
4	30 ± 2 (48 ± 5)	0 (2.8 ± 0.4)	41 ± 2 (67 ± 7)	0 (3.4 ± 0.3)
5	96 ± 5	0 (6.1 ± 0.7)	91 ± 4	3 ± 1 (7.4 ± 0.9)
6	78 ± 4	5 ± 1 (4.8 ± 0.4)	77 ± 3	8 ± 1 (7.9 ± 0.6)
7	98 ± 4	100 ± 5	99 ± 5	96 ± 4
8	100 ± 5	96 ± 4	98 ± 5	98 ± 4
9	85 ± 4	23 ± 2 (49.4 ± 5.1)	87 ± 4	28 ± 1 (55.2 ± 6.3)
10	98 ± 4	98 ± 4	99 ± 5	100 ± 5
11	100 ± 5	100 ± 5	96 ± 4	93 ± 4
12	98 ± 4	100 ± 5	97 ± 4	90 ± 4
13	97 ± 4	98 ± 5	96 ± 4	72 ± 3
14	100 ± 4	94 ± 4	98 ± 5	89 ± 5
fosfomycin	N.d.	0 (0.21 ± 0.04)	N.d.	0 (0.30 ± 0.05)

RA: residual activity. Residual activities were determined at 100 µM. ^a^: time of preincubation = 0 min. ^b^: time of preincubation: 10 min. N.d.: not determined.

## Data Availability

Data is contained within the article and Supplementary Materials.

## Supplementary Materials

The following are available online at https://www.mdpi.com/article/10.3390/antibiotics10121535/s1, Figure S1: Docking results. Scatterplots and boxplots of Glide and Hybrid scores, Figure S2: Glide scores per compound and each enzyme analyzed, Figures S3–S8: 1H-NMR and 13C-NMR spectra of compounds 5, 9 and 10, Figures S9–S11: ESI-ITMS spectra of compounds **5**, **9** and **10**.
