# Supplementary material for: Inhibition of MurA Enzyme from Escherichia coli and Staphylococcus aureus by Diterpenes from Lepechinia meyenii and Their Synthetic Analogs
Source: Antibiotics (Basel). 2021 Dec 15;10(12):1535. doi: 10.3390/antibiotics10121535 (PMC8698320; doi:10.3390/antibiotics10121535)
Supplement: Supplementary file 1 [file antibiotics-10-01535-s001.zip › antibiotics-1484460-supplementary.pdf]

antibiotics

MDPI

Article

Inhibition of MurA enzyme from *Escherichia coli* and *Staphylococcus aureus* by diterpenes from *Lepechinia meyenii* and their synthetic analogs

Macarena Funes Chabán [1,†], Martina Hrast [2,†], Rok Frlan [2,†], Dafni G. Graikioti [3], Constantinos M. Athanassopoulos [3] and María Cecilia Carpinella*[,1]

[1] Fine Chemical and Natural Products Laboratory, IRNASUS CONICET-UCC, Universidad Católica de Córdoba, Córdoba X5016DHK, Argentina; macarenafuneschaban@ucc.edu.ar (M. F-Ch.)
[2] Faculty of Pharmacy, University of Ljubljana, SI-1000 Ljubljana, Slovenia; martina.hrast@ffa.uni-lj.si (M.H.); Rok.Frlan@ffa.uni-lj.si (R.F.).
[3] Synthetic Organic Chemistry Laboratory, Department of Chemistry, University of Patras, Greece; dafnigraikioti@upnet.gr (D.G.G.); kath@chemistry.upatras.gr (C.M.A.)
* Correspondence: ceciliacarpinella@ucc.edu.ar; Tel.: +543514938000

Supplementary Materials

NMR spectra

^{1}H NMR spectra were obtained at 600.13 MHz and ^{13}C NMR spectra at 150.90 MHz on a Bruker AVANCEIII HD spectrometer.

ESI-ITMS spectra

ESI-ITMS spectra were obtained on a Bruker AmaZon SL spectrometer at negative ion mode.

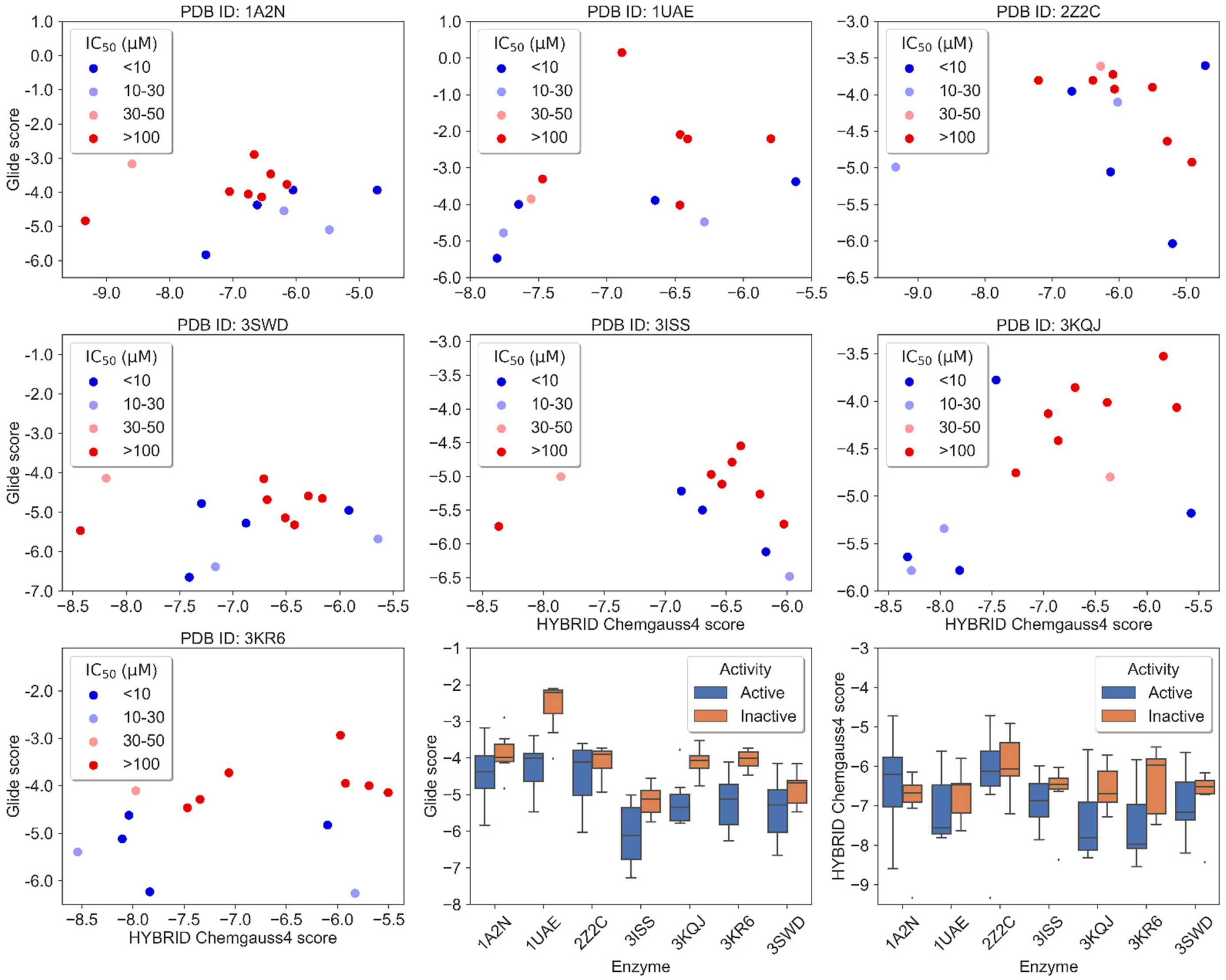

Figure S1. Docking results. Scatterplots of Glide and Hybrid scores for each compound and each enzyme used; boxplots of Hybrid and Glide docking scores of active and inactive compounds calculated for each enzyme separately.

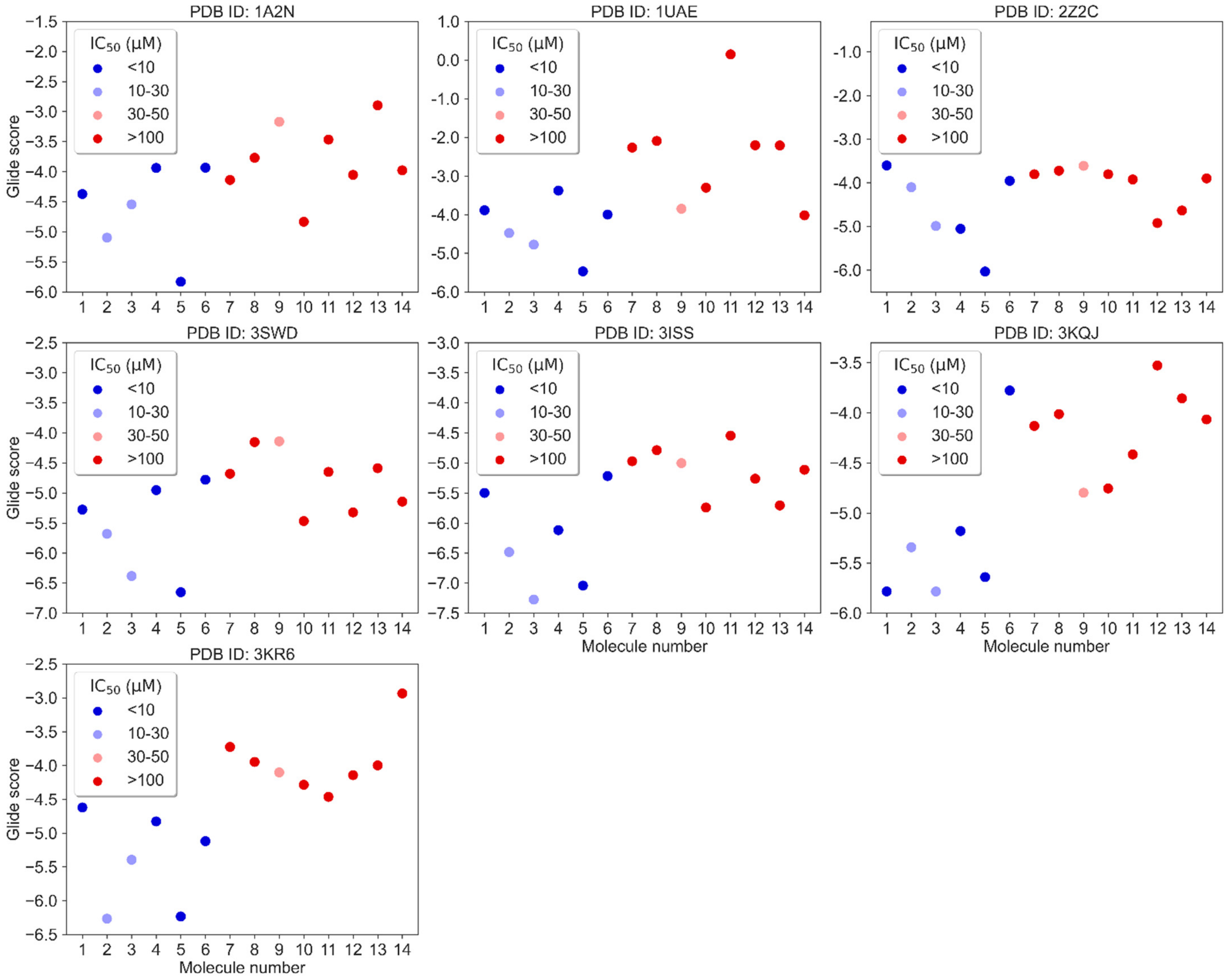

Figure S2. Glide scores per compound and each enzyme analyzed.

NMR spectra data

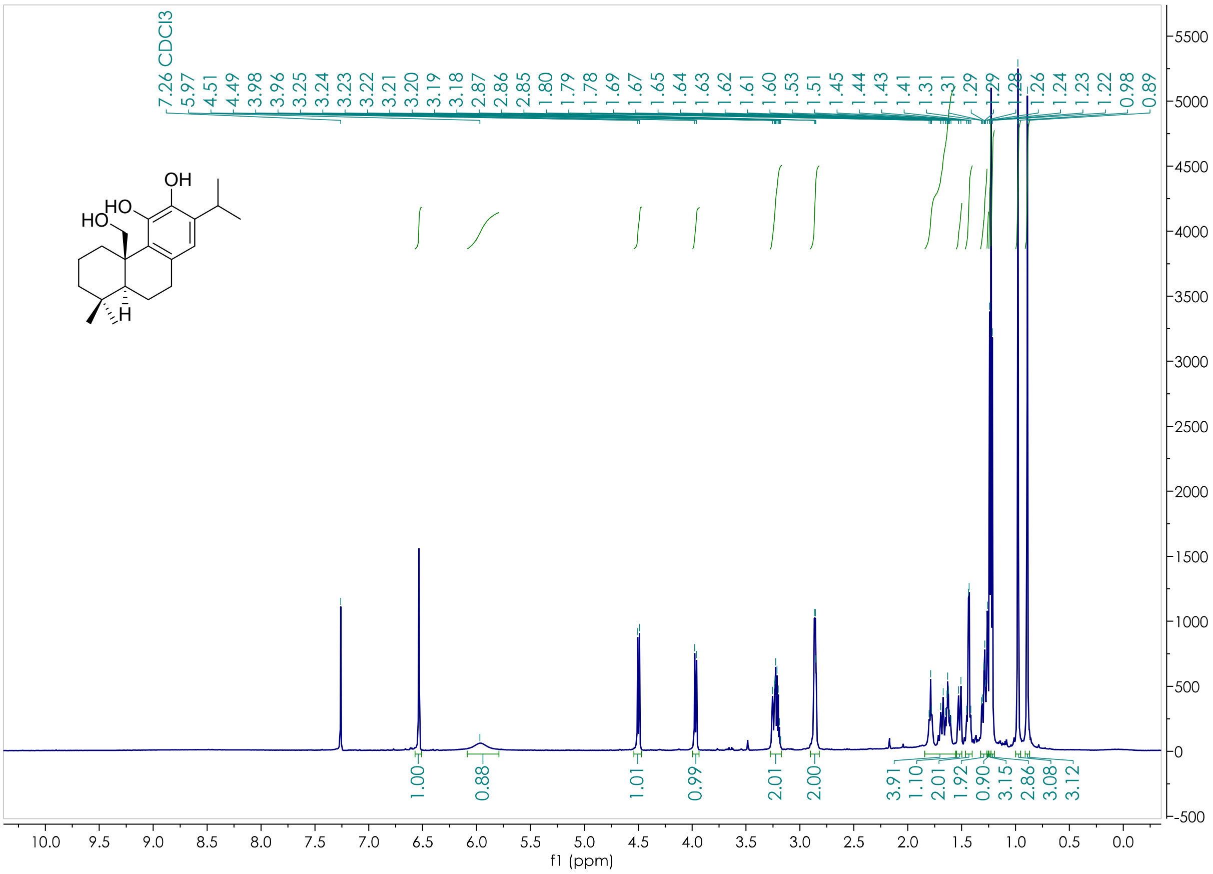

Figure S3. ***^{1}H-NMR spectrum of compound 5***

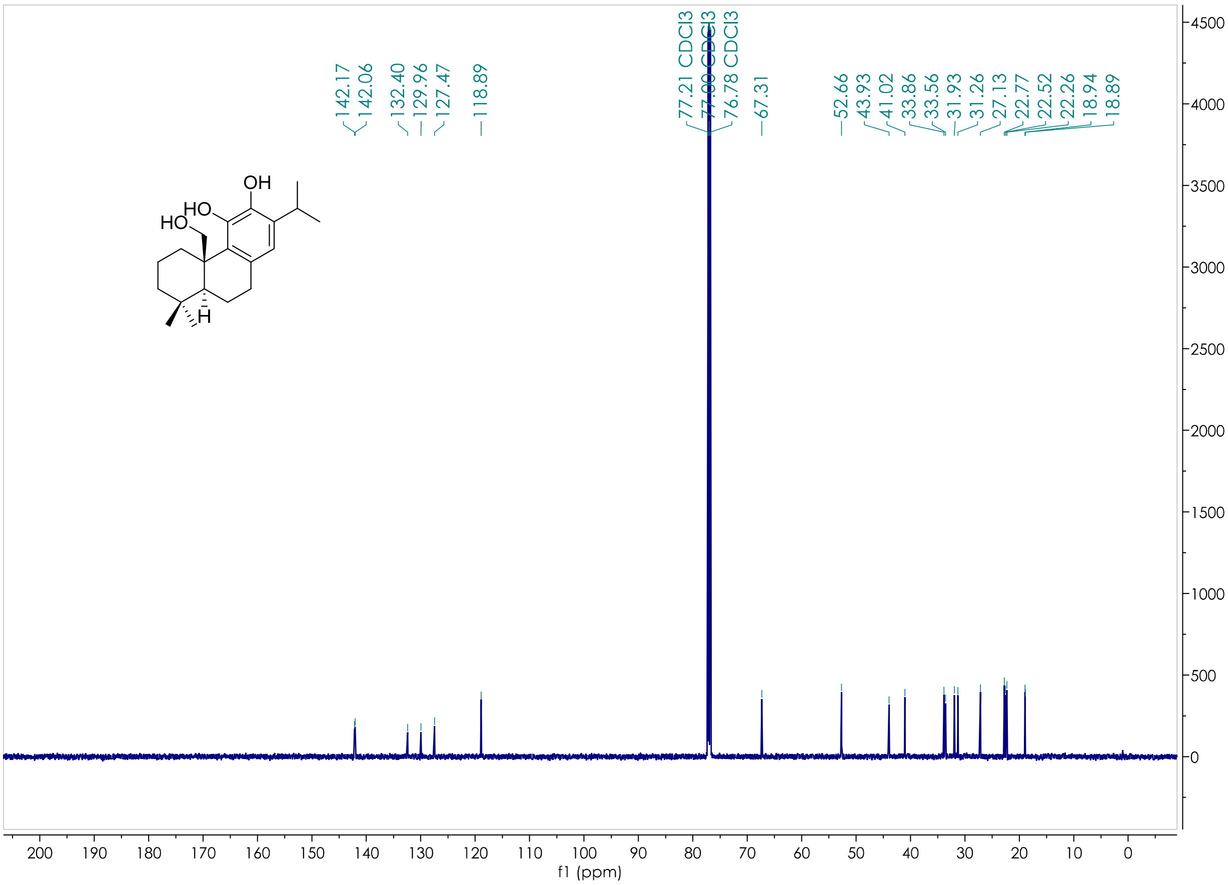

Figure S4. ***^{13}C-NMR spectrum of compound 5***

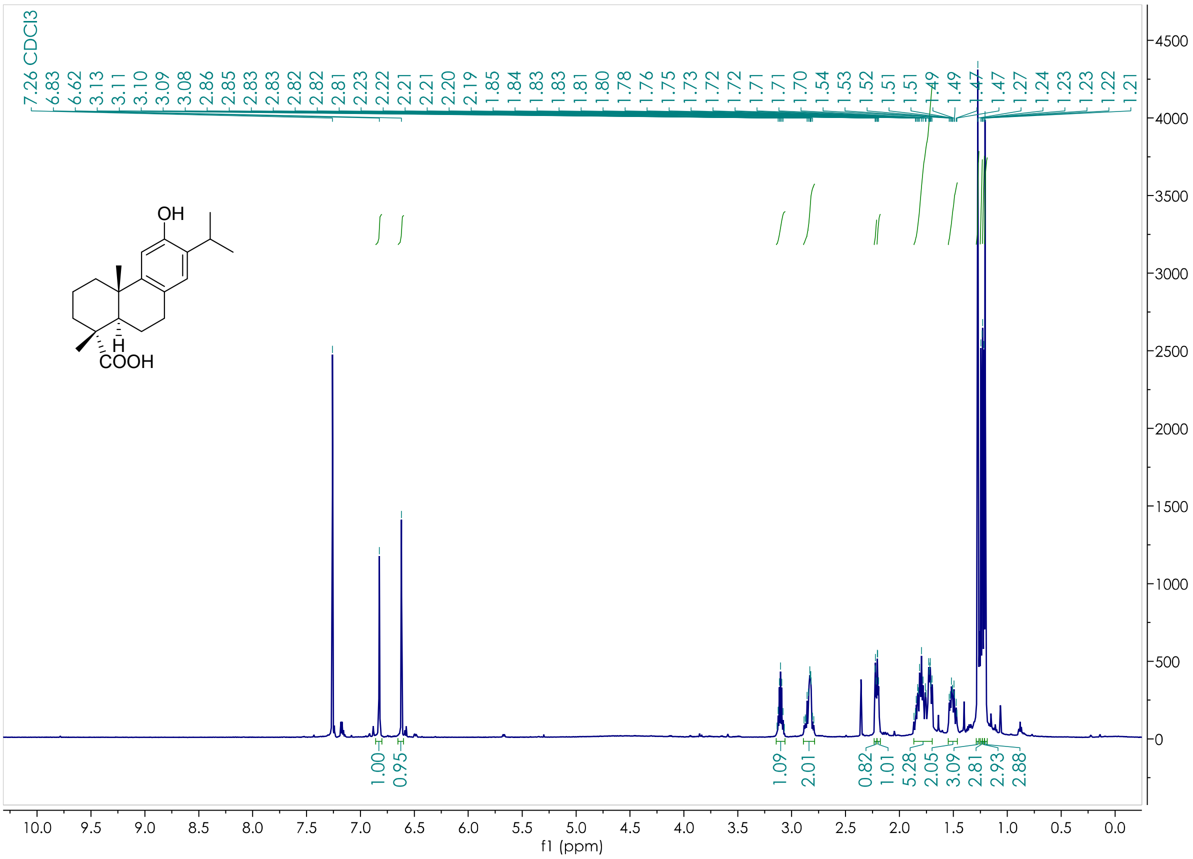

Figure S5. [1]**H-NMR spectrum of compound 9**

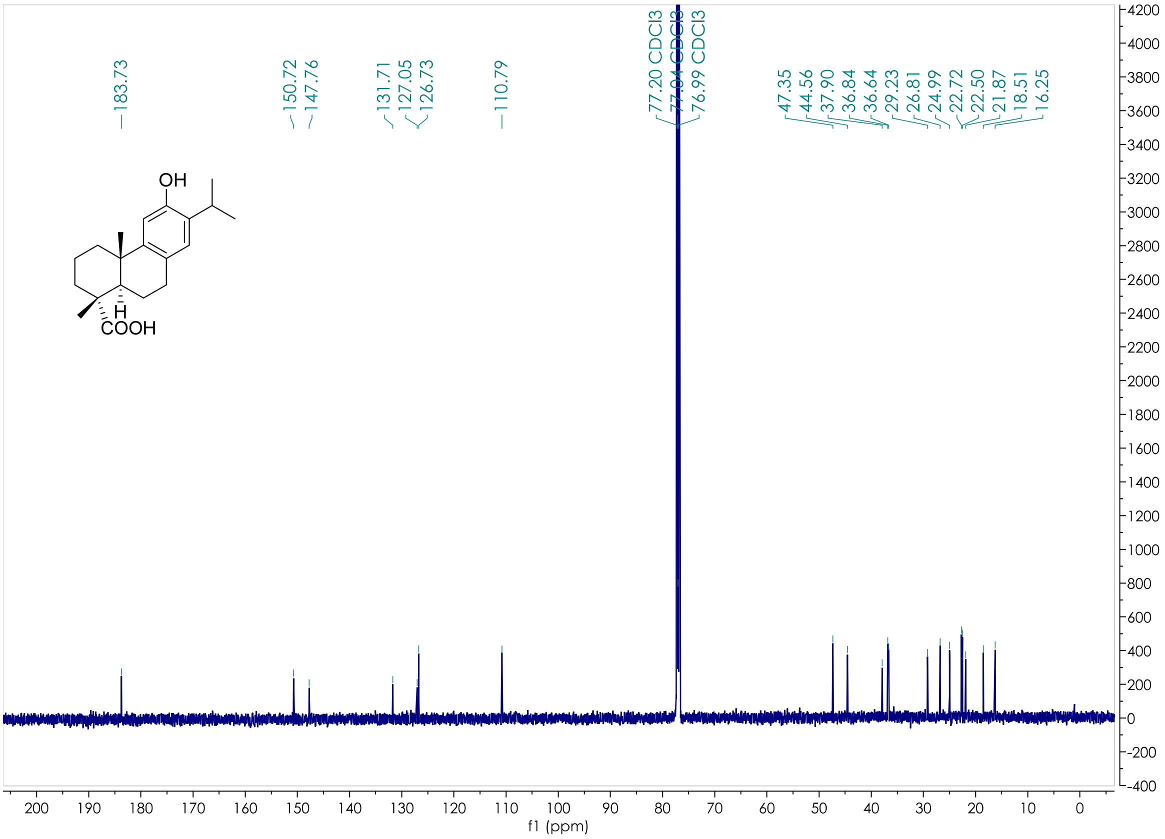

Figure S6. [13]**C-NMR spectrum of compound 9**

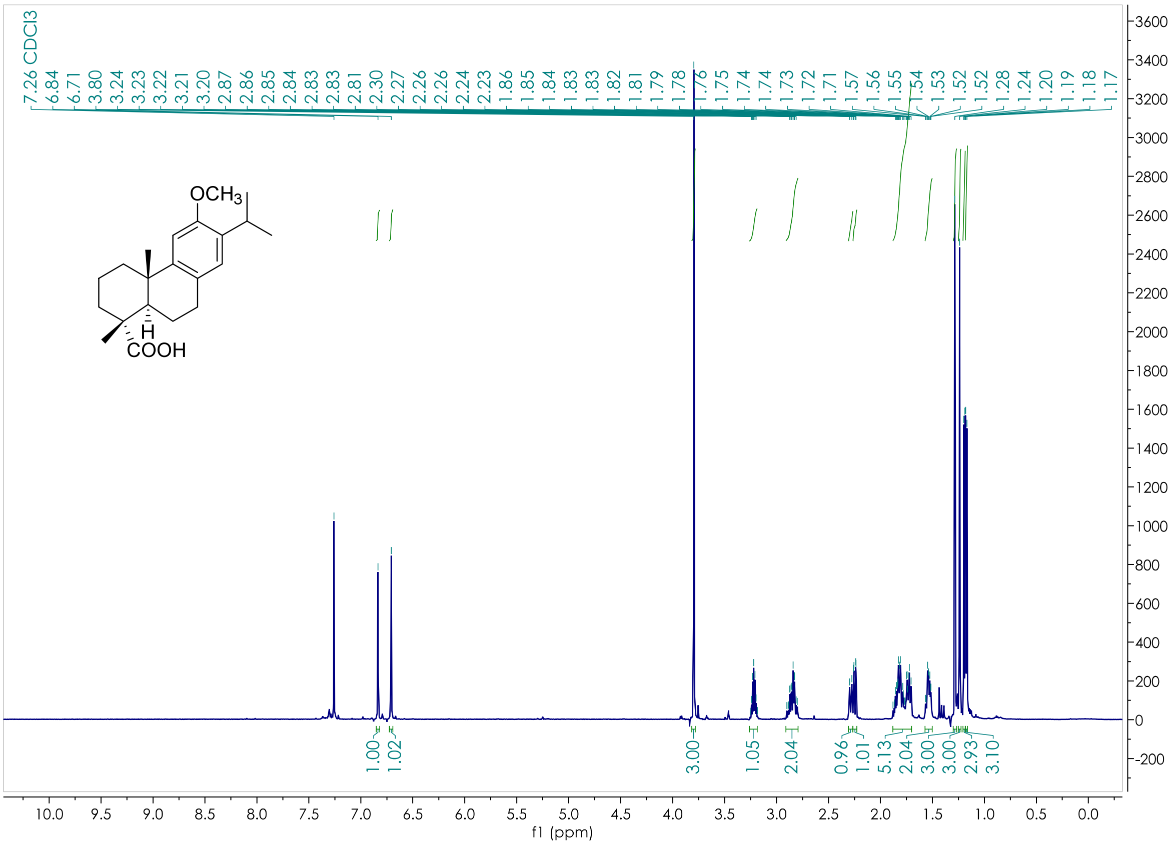

Figure S7. ***¹H-NMR spectrum of compound 10***

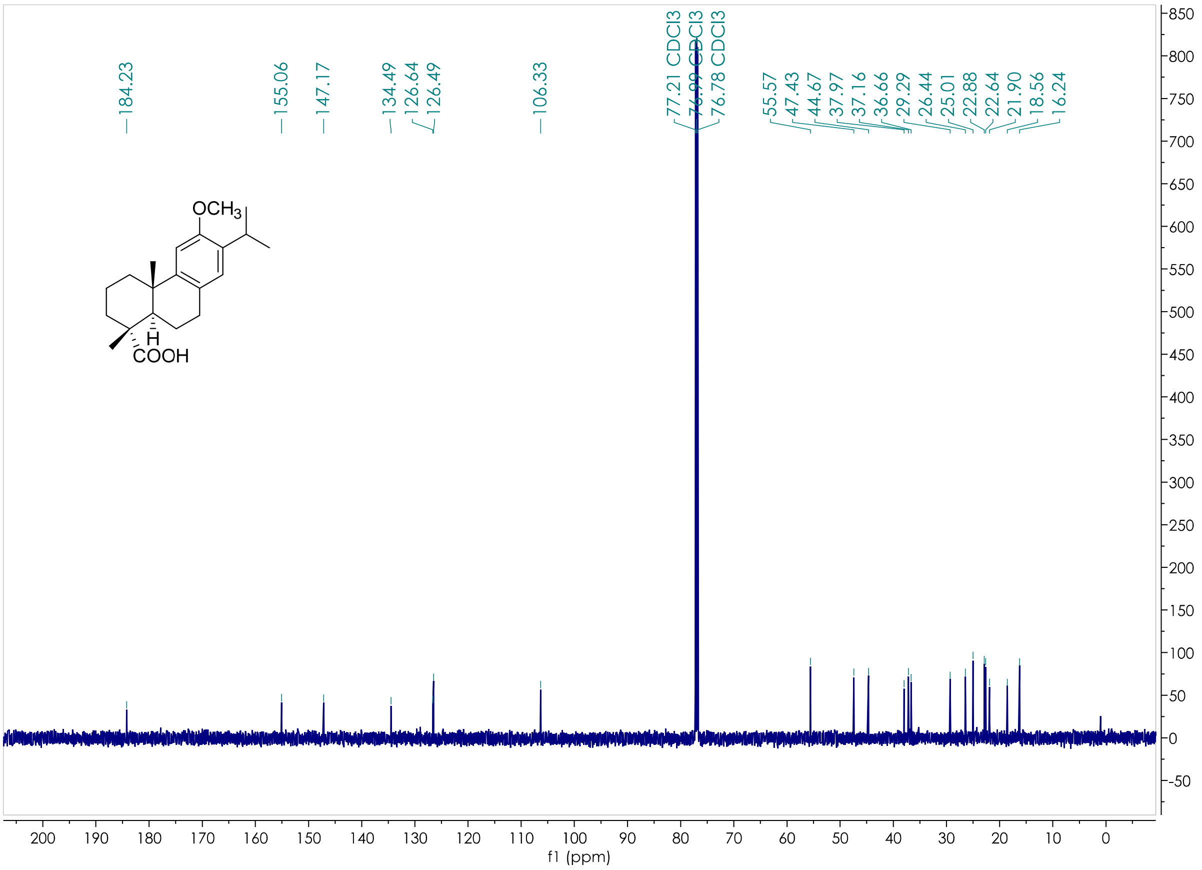

Figure S8. ***¹³C-NMR spectrum of compound 10***

MS spectra data

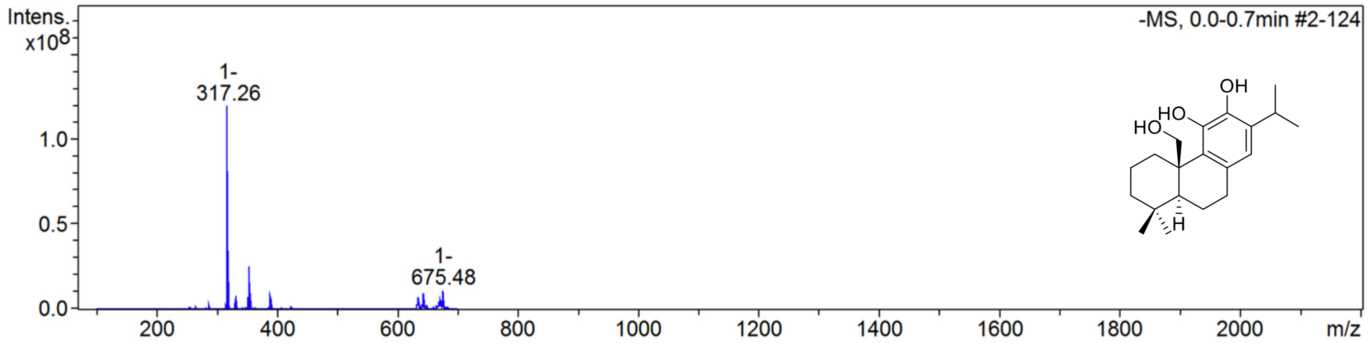

Figure S9. ESI-ITMS spectrum of compound 5

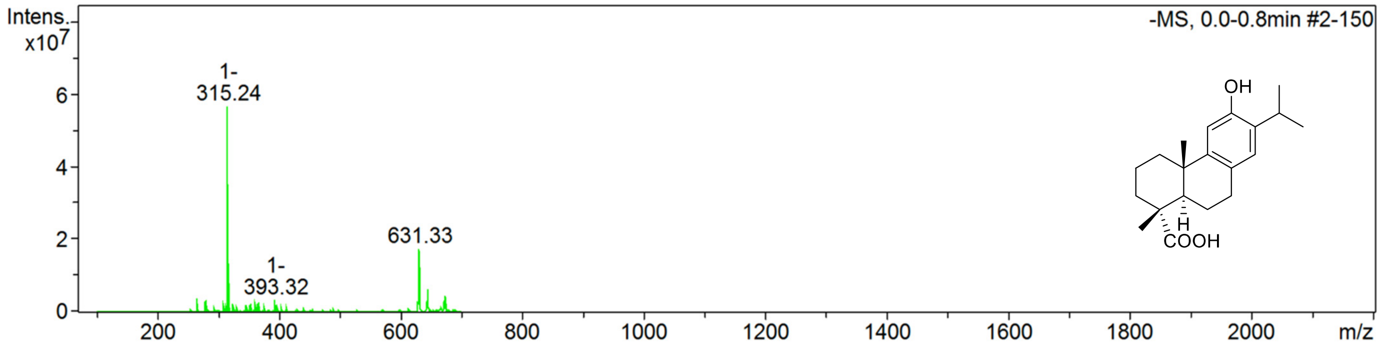

Figure S10. ESI-ITMS spectrum of compound 9

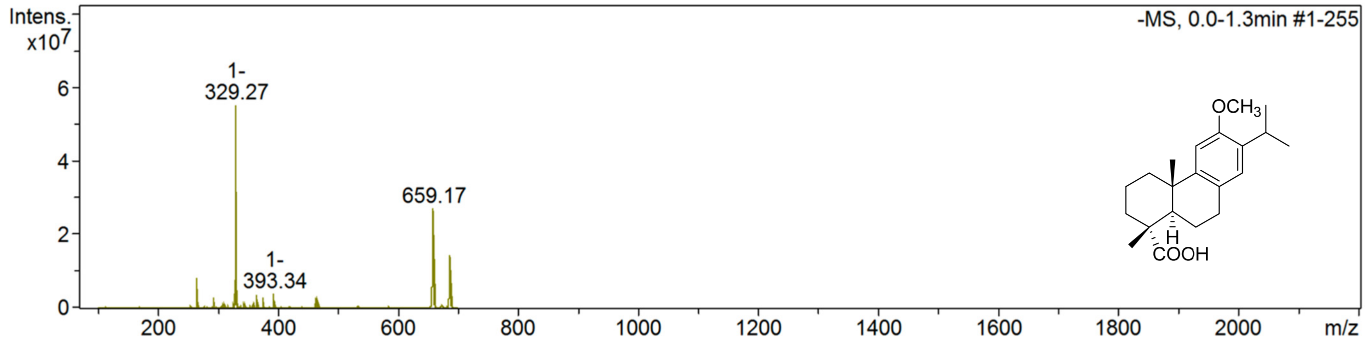

Figure S11. ESI-ITMS spectrum of compound 10
